# A Bifunctional Anti-Amyloid Blocks Oxidative Stress and the Accumulation of Intraneuronal Amyloid-Beta

**DOI:** 10.3390/molecules23082010

**Published:** 2018-08-12

**Authors:** Silvia Hilt, Robin Altman, Tamás Kálai, Izumi Maezawa, Qizhi Gong, Sebastian Wachsmann-Hogiu, Lee-Way Jin, John C. Voss

**Affiliations:** 1 Department of Biochemistry & Molecular Medicine, University of California, Davis, CA 95616, USA; slhilt@ucdavis.edu; 2 Department of Biological Sciences, California State University Sacramento, Sacramento, CA 95819, USA; altman@csus.edu; 3 Institute of Organic and Medicinal Chemistry, University of Pécs, H-7624 Pécs, Szigeti st. 12., H-7624 Pécs, Hungary; tamas.kalai@aok.pte.hu; 4 Medical Investigation of Neurodevelopmental Disorders (M.I.N.D.) Institute and Department of Pathology and Laboratory Medicine, University of California, Davis, Sacramento, CA 95817, USA; imaezawa@ucdavis.edu (I.M.); lwjin@ucdavis.edu (L.-W.J.); 5 Department of Cell Biology and Human Anatomy, School of Medicine, University of California, Davis, Davis, CA 95616, USA; qzgong@ucdavis.edu; 6 Department of Pathology and Laboratory Medicine, and Center for Biophotonics, University of California Davis, Sacramento, CA 95817, USA; sebastian.wachsmannhogiu@mcgill.ca; 7 Department of Bioengineering, McGill University, Montreal, QC H3A OE9, Canada

**Keywords:** amyloid beta, Alzheimer’s disease, bifunctional drug, Aβ oligomer, oxidative stress, intraneuronal Aβ, intracellular Aβ, nitroxide antioxidant, spin label

## Abstract

There is growing recognition regarding the role of intracellular amyloid beta (Aβ) in the Alzheimer’s disease process, which has been linked with aberrant signaling and the disruption of protein degradation mechanisms. Most notably, intraneuronal Aβ likely underlies the oxidative stress and mitochondrial dysfunction that have been identified as key elements of disease progression. In this study, we employed fluorescence imaging to explore the ability of a bifunctional small molecule to reduce aggregates of intracellular Aβ and attenuate oxidative stress. Structurally, this small molecule is comprised of a nitroxide spin label linked to an amyloidophilic fluorene and is known as spin-labeled fluorene (SLF). The effect of the SLF on intracellular Aβ accumulation and oxidative stress was measured in MC65 cells, a human neuronal cell line with inducible expression of the amyloid precursor protein and in the N2a neuronal cell line treated with exogenous Aβ. Super-resolution microscopy imaging showed SLF decreases the accumulation of intracellular Aβ. Confocal microscopy imaging of MC65 cells treated with a reactive oxygen species (ROS)-sensitive dye demonstrated SLF significantly reduces the intracellular Aβ-induced ROS signal. In order to determine the contributions of the separate SLF moieties to these protective activities, experiments were also carried out on cells with nitroxides lacking the Aβ targeting domain or fluorene derivatives lacking the nitroxide functionality. The findings support a synergistic effect of SLF in counteracting both the conformational toxicity of both endogenous and exogenous Aβ, its promotion of ROS, and Aβ metabolism. Furthermore, these studies demonstrate an intimate link between ROS production and Aβ oligomer formation.

## 1. Introduction

Alzheimer’s disease (AD) is a neurodegenerative disorder characterized by progressive and irreversible cognitive decline. Hallmarks of AD pathology include the deposition of extracellular insoluble amyloid beta (Aβ) plaques and hyper-phosphorylation of tau protein, which lead to intracellular accumulation of neurofibrillary tangles (NFT). More recently, however, stronger emphasis has been placed on two interdependent initiators of the neurodegenerative cascade in AD: accumulation of small, soluble, intracellular Aβ oligomers (AβO) and the subsequent AβO-induced neuro-inflammation that leads to the formation of reactive oxygen species (ROS) and cellular damage [1,2,3].

Numerous mechanisms have been proposed to account for Aβ toxicity since the peptide has been found to interact with and/or dysregulate a plethora of cellular systems. While most experimental and clinical efforts have focused on extracellular Aβ, there is growing evidence supporting a significant role for intracellular oligomeric AβO in the etiology of AD [4,5]. Intraneuronal Aβ precedes both intracellular NFTs and extracellular amyloid deposits [6] and has profound effects on neuronal health [7]. For example, intracellular Aβ was found to be considerably more toxic than extracellular Aβ [5,8]. The two pools of Aβ seem to exist in a dynamic equilibrium [9], which means targeting intracellular Aβ may also influence the levels of extracellular AβO. 

Many studies report oxidative stress plays a major role in AD progression. Oxidative stress occurs when there is a physiological imbalance between a high level of ROS and the body’s ability to produce enough ROS scavengers to counteract the ROS [10,11,12]. While oxidative stress is a major indicator of aging, increased levels of oxidative stress markers are present in the brains of patients in different stages of AD. For example, lipid peroxidation marker levels are consistently elevated during the early stages of AD when compared to patients with mild cognitive impairment (MCI), late stage AD, or those with Parkinson’s disease [13]. In addition to the modification of lipids, proteins, and DNA, lipid peroxidation byproducts produced during oxidative stress damage mitochondria and up-regulate tau phosphorylation [14,15]. Although the mechanism of the ROS cascade in AD is unclear, Aβ is an efficient generator of hydrogen peroxide [16], which can lead to mitochondrial impairment and further generation of ROS [17]. Aβ may also interact directly with the respiration chain of mitochondria, resulting in dysfunction and elevated ROS production [18]. This is consistent with clinical observations correlating mitochondrial oxidative damage with disease progression in AD patients [12,19].

**Figure 1:**
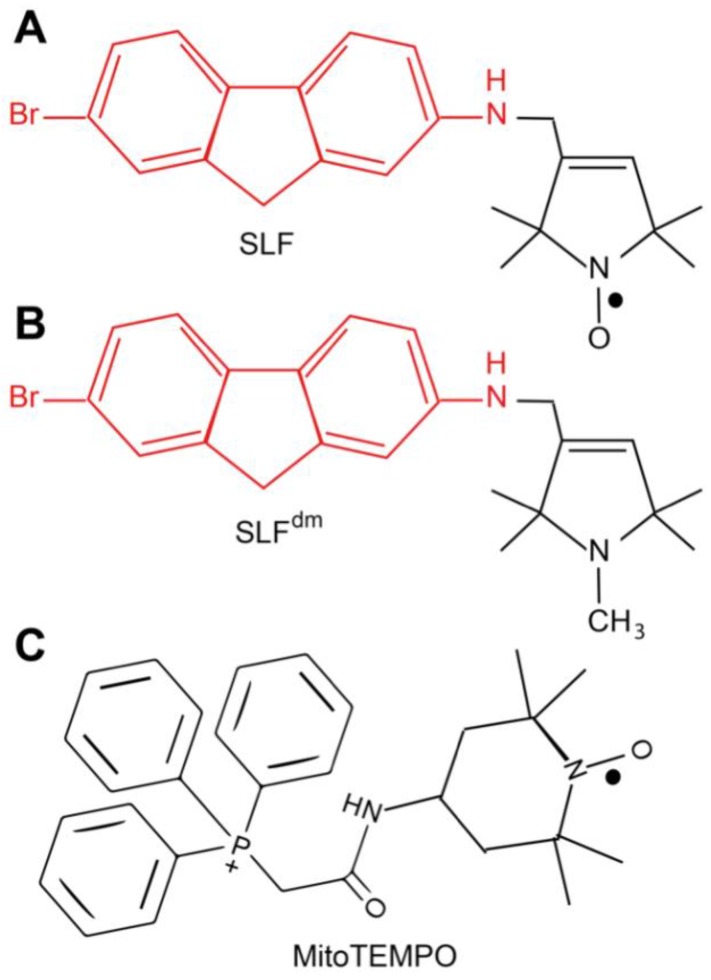
(**A**) The chemical structure of the spin-labeled fluorene (SLF). The amyloid-targeting domain is shown in red and the nitroxide spin label moiety is shown in black. The diamagnetic form of SLF (SLF^dm^) that lacks the nitroxyl antioxidant capacity is shown in (**B**). (**C**) The structure of the antioxidant MitoTEMPO.

We have, therefore, focused our efforts on designing targeted bifunctional compounds that convert AβO into a nontoxic state and also block AβO-induced cellular oxidative damage. We have previously described one such compound known as the spin-labeled fluorene (SLF, Figure 1) [20,21,22]. SLF is comprised of a fluorene core that binds to Aβ with high affinity [22] and stabilizes AβO into small nontoxic oligomers [20,23]. The bifunctionality of SLF arises from its nitroxide spin label moiety, which provides catalytic antioxidant activity [24,25]. The ability of sterically hindered cyclic nitroxides to serve as mimics of superoxide dismutase within cells has led to their consideration as anti-cancer [26], radioprotective [27], and anti-aging [28] agents. In this study, we use confocal and super-resolution microscopy to demonstrate that SLF blocks Aβ-induced ROS generation within cultured neurons, inhibits intraneuronal oligomer formation, and facilitates clearance of intraneuronal Aβ. Furthermore, by evaluating the functional components of SLF independently, we provide new insights on the interdependence of oxidative stress and AβO toxicity. 

## 2. Results

### 2.1. In Cultured Neurons, the Bifunctional Activity of SLF Offers Superior Protection against the Toxicity of both Exogenous Aβ and Aβ Peptide Generated Intracellularly

**Figure 2:**
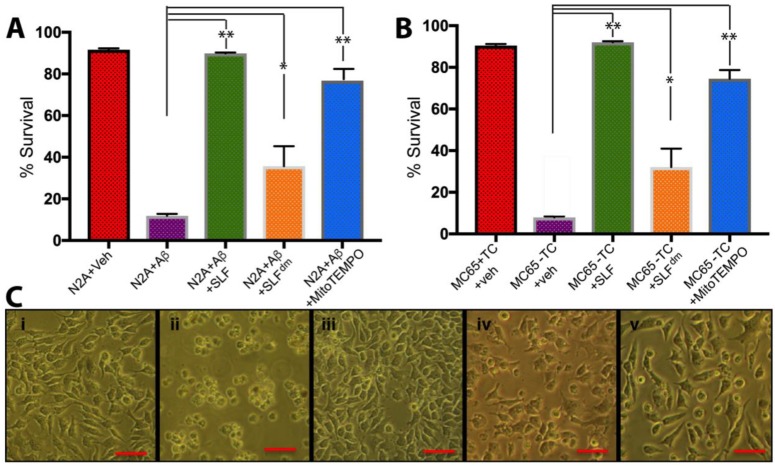
Protection against Aβ toxicity by SLF compared to agents bearing only an antioxidant or anti-amyloid activity. Neuronal viability by the MTT assay was determined for exogenous Aβ (N2a cells) and endogenous Aβ (MC65 cells) after 72 hours. (**A**) 1 μM exogenous Aβ was added to cultured N2a neurons and cell viability was compared to the +Aβ group. The effect of 2 μM SLF, SLF^dm^, and MitoTEMPO co-addition on the survival of AβO-treated N2a cells is given by the green, orange, and blue bars, respectively. The ability of SLF and its independent functionalities to protect against Aβ toxicity in MC65 neurons containing Tet-Off inducible gene expression for the Aβ precursor (APP) is shown in (**B**). The viability of the non-induced (+TC) cells is given by the red bar. The effect of 2 μM SLF, SLF^dm^ and MitoTEMPO addition to the APP-induced (−TC) cell survival is given by the green, orange, and blue bars, respectively, and is compared to the −TC group. For both (**A**) and (**B**), compounds were added upon APP induction and cell survival was measured 72 hours later. Statistical analyses of cell counts by one-way ANOVA gives * *p* < 0.01, ** *p* < 0.001, *n* = 9. Error bars represent the standard error as described in the Methods section. Panel (**C**) shows light microscopy images of MC65 cell cultures three days without APP induction (**i**), with APP induction (**ii**), with APP induction in the presence of 2 μM SLF (**iii**), with APP induction in the presence of 2 μM SLF^dm^ (**iv**), and with APP induction in the presence of 2 μM MitoTEMPO (**v**).

We have previously shown that SLF is a more potent protector against Aβ toxicity compared to the parent fluorene molecule lacking the nitroxide spin label moiety [21]. Both SLF and the parent fluorene bind AβO, reverse the peptide aggregation, and block Aβ toxicity [20,21,22,23]. In order to further evaluate the significance of the antioxidant functionality in SLF protection, we compared the ability of SLF, a diamagnetic version of SLF (SLF^dm^), and MitoTEMPO, which is a potent mitochondrial-targeted nitroxide antioxidant, to counteract Aβ toxicity in cultured N2a neurons (Figure 2A). After reaching 80% confluence, the cultured cells were co-treated with Aβ and with either the vehicle (DMSO) or the aforementioned compounds to a final concentration of 1 μM and 2 μM, respectively. The cultured cells were incubated at 37 °C for 24 hours. The MTT assay was performed to measure cell survival and one-way ANOVA data on the percentage survival of cells show that when SLF is added to N2a cells treated with exogenous Aβ, there is about an eight-fold increase in cell survival (Figure 2A) compared to cells treated with exogenous Aβ alone (Figure 2A). Comparatively, the SLF^dm^ increases cell survival by only about three-fold, which highlights the significance of the nitroxyl moiety in ROS protection (Figure 2A). The MitoTEMPO compound that has an ROS scavenger moiety similar to SLF also increases cell viability by about seven-fold (Figure 2A). Similarly, we compared the protection of the SLF moieties in the human neuroblastoma MC65 cell line, which generates large amounts of intraneuronal Aβ by overexpressing the amyloid precursor protein (APP) through Tet-Off transcriptional regulation [29] (Figure 2B). Control cells were grown with tetracycline (+TC, Figure 2B) until the cells reached 80% confluence, at which point the Aβ challenge was initiated by removing tetracycline from the culture media. The compounds (SLF, SLF^dm^, and MitoTEMPO) were added to a final concentration of 2 μM. The cells were incubated at 37 °C for 72 hours, which was followed by MTT analysis of cell survival. Statistical analysis shows SLF exerts maximum protection in cells overexpressing APP precursor protein and has about a 14-fold increase in cell survival (Figure 2B) over APP-induced cells (Figure 2B). SLF^dm^ increases cell survival by about five-fold (Figure 2B) while the MitoTEMPO compound increases cell survival by about 10-fold (Figure 2B). Overall, the cell survival assay showed a similar pattern for protection against both exogenous and endogenous Aβ with SLF inducing the most protection, followed by MitoTEMPO and SLF^dm^ (Figure 2A and Figure 2B). The effect of APP induction and compound treatment on MC65 cell morphology is shown in Figure 2C. MC65 cells grown in the absence of APP induction (+TC) maintain cell morphology at three days while MC65 cells induced for APP expression (−TC) exhibit 100% alteration of cell morphology. APP-induced cells in the presence of 2 μM SLF show unaltered cell morphology, which is indicative of SLF’s ability to protect neurons from Aβ-induced cellular damage. In contrast, both SLF^dm^ and MitoTEMPO offer limited protection of neuronal morphology.

### 2.2. SLF’s Nitroxide Component Plays a Key Role in Decreasing Aβ-Induced Oxidative Stress in a Human Neuroblastoma Cell Line (MC65) Overexpressing the Amyloid Precursor Protein

**Figure 3:**
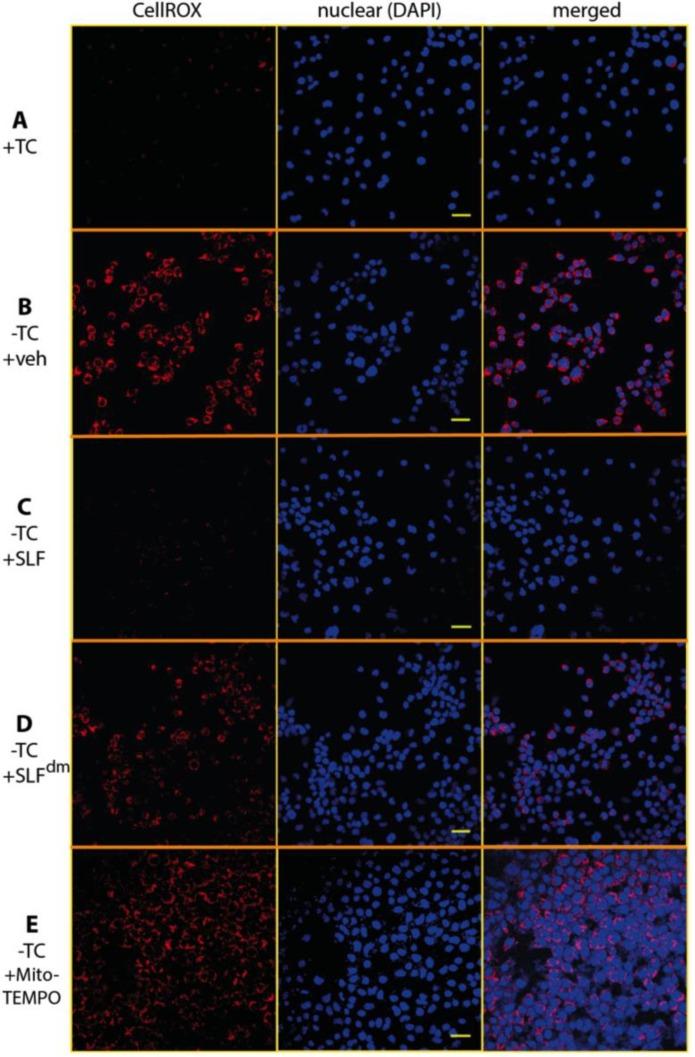
The nitroxide moiety of SLF has extensive ROS scavenging properties in cultured neuronal cells induced to overexpress the amyloid precursor protein (APP). Confocal microscopy images show Aβ-induced ROS signal reported by the fluorogenic dye CellRox Deep Red (red punctae in image) in MC65 human neuroblastoma cells when APP expression is turned on (**B**) relative to the control (**A**). In cells that are overexpressing APP, SLF greatly attenuates the ROS signal (**C**). SLF lacking the nitroxyl moiety (**D**) and the MitoTEMPO antioxidant (**E**) provide lower ROS scavenging activity compared to SLF. In addition to the CellROX images (left column), the DAPI nuclear stain (middle column) and the merged DAPI-CellRox images (right column) are shown. Scale bar represents 20 μm.

**Figure 4:**
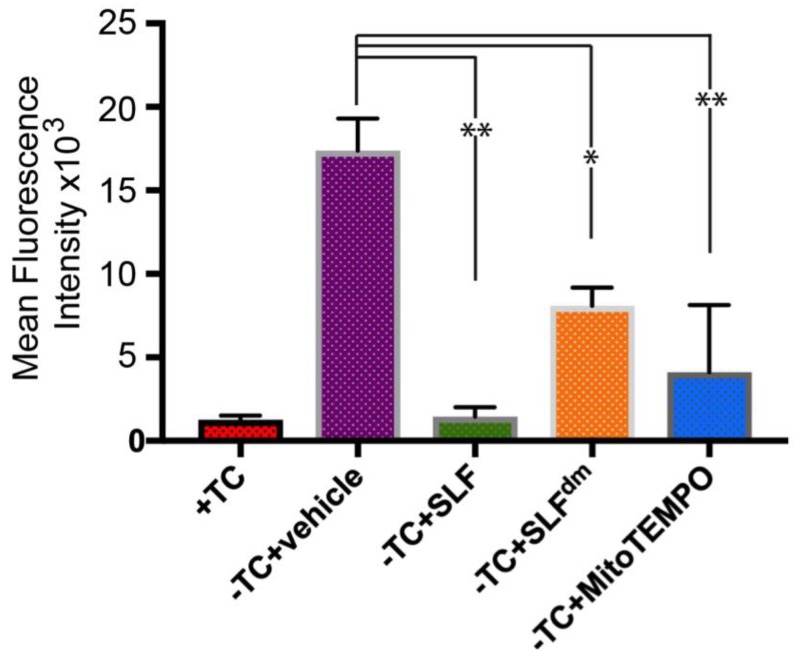
Quantification of mean fluorescence intensity signal of Aβ-induced ROS signal (see Figure 3) in human neuronal cells overexpressing the amyloid precursor protein (APP). The effect on Aβ-induced ROS signal of SLF, SLF^dm^, and MitoTEMPO addition to the APP-induced cells (−TC) is given by the green, orange, and blue bars, respectively, and is compared to the −TC group. Statistical analyses of fluorescence intensity by one-way ANOVA gives * *p* < 0.01, ** *p* < 0.001 for *n* = 3. Error bars represent the standard error as described in the Methods section.

The role of Aβ in increasing oxidative stress has been well-documented using various methods to detect reactive oxidative species [30,31,32]. To determine if treatment with SLF attenuates Aβ-induced ROS production, we cultured the MC65 neurons in the presence and absence of SLF upon induction of the Aβ precursor, APP. Intracellular Aβ is known to start accumulating as early as 4 hours after TC removal in the MC65 cell line and most unprotected cells die after three days. In order to avoid the detection of oxidative changes due to cell death toxicity, we imaged cells stained with the ROS-sensitive dye CellROX at the 24–hour time period [33]. As shown in Figure 3B, expression-induced cells show a clear red CellROX signal, which indicates a high level of oxidative stress. When APP-expressing cells are treated with SLF, ROS levels are significantly lowered (Figure 3C). In order to confirm the role of the nitroxide spin label moiety in attenuating Aβ-induced oxidative stress, we also treated APP-expressing cells with the diamagnetic version of SLF (SLF^dm^), which lacks the catalytic antioxidant functionality. As shown in Figure 3D, SLF^dm^ only partially lowers ROS levels relative to the vehicle control. The significance of the nitroxide moiety alone is confirmed by the ability of the nitroxide-based antioxidant MitoTEMPO to attenuate oxidative stress in Aβ-challenged neurons (Figure 3E). Quantification of CellROX intensities is given in Figure 4. The superior performance of SLF (Figure 4) in lowering oxidative stress suggests its ability to provide a targeted antioxidant activity that underlies its potency in protecting against Aβ toxicity.

### 2.3. The Nitroxide Group of the SLF Compound Plays a Key Role in Decreasing Exogenous Aβ-Induced Oxidative Stress

**Figure 5:**
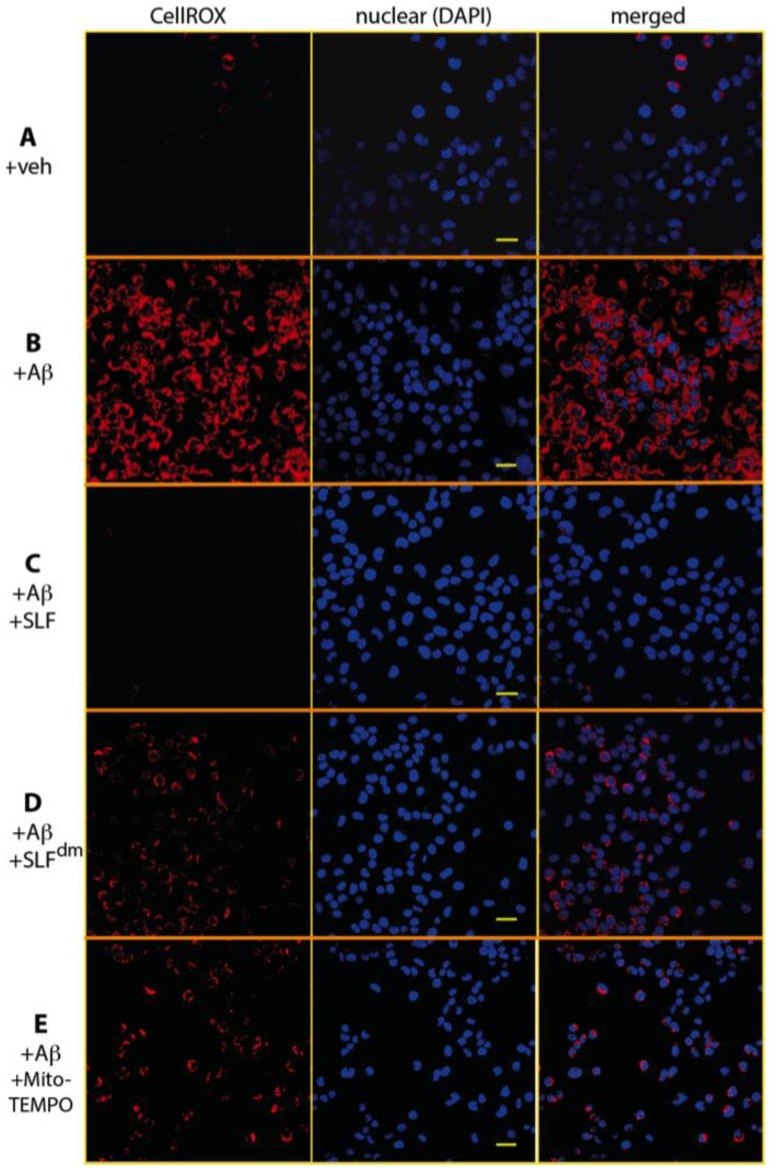
The nitroxide moiety of SLF has extensive ROS scavenging properties in N2a cells treated with 1 μM exogenous Aβ. ROS levels were imaged by confocal microscopy for the fluorogenic dye CellRox Deep Red (red punctae in image). A slight generation of ROS is evident with the vehicle control (**A**). Addition of Aβ elicits a strong ROS response (**B**). Images show decreased levels of the Aβ-induced ROS signal in the presence of SLF (**C**) while moderately high levels of ROS are evident in cells treated with the SLF lacking the nitroxyl moiety (**D**). The strong antioxidant activity of MitoTEMPO is evident in (**E**). In addition to the CellROX images (left column), the DAPI nuclear stain (middle column) and merged DAPI-CellRox images (right column) are shown. Scale bar represents 20 μm.

**Figure 6:**
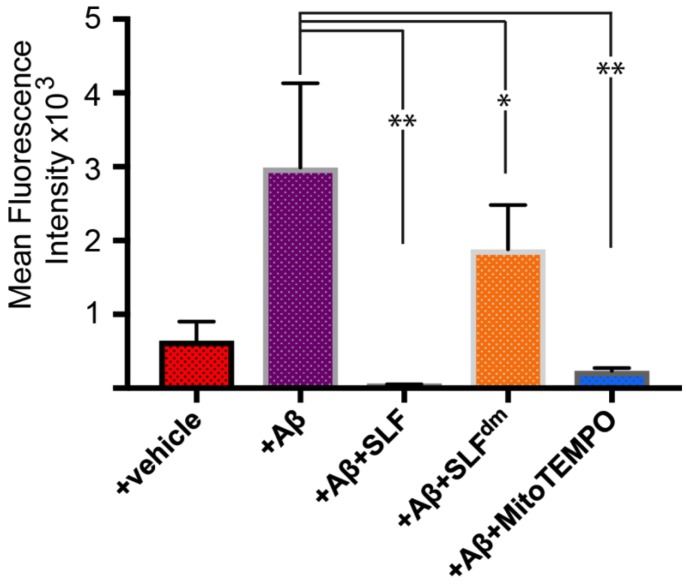
Quantification of mean fluorescence intensity signal from Aβ-induced ROS signal (see Figure 5) in N2a neuronal cells treated with 1 μM exogenous Aβ. The effect of SLF, SLF^dm^, and MitoTEMPO co-addition on the Aβ-induced ROS signal of Aβ-treated N2a cells is given by the green, orange, and blue bars, respectively, and is compared to the +Aβ group. Statistical analysis of fluorescence intensity by one-way ANOVA gives * *p* < 0.01, ** *p* < 0.001, *n* = 3. Error bars represent the standard error as described in the Methods section.

To determine the ability of SLF to attenuate oxidative stress from exogenous Aβ, we first measured AβO-induced oxidative stress in N2a cultured neurons (Figure 5). We then evaluated the abilities of SLF, MitoTEMPO, and SLF^dm^ to attenuate the oxidative stress resulting from an exogenous AβO challenge. We co-treated N2a cells with the compounds (2 μM final concentration) and exogenous Aβ (1 μM final concentration), incubated the cultures for 24 hours at 37 °C, and then imaged the CellROX signal. Both SLF and MitoTEMPO strongly inhibit ROS generation in Aβ-challenged neurons (Figure 5C and Figure 5E). SLF^dm^ offers partial protection against ROS generation, which suggests that conformational modulation of Aβ by the fluorene moiety can regulate the ability of Aβ to promote oxidative stress. A quantification of ROS signal in the N2a cells is given in Figure 6.

### 2.4. SLF Attenuates the Cytoplasmic Accumulation of Intracellular Aβ in a Human Neuroblastoma Cell Line Overexpressing the Amyloid Precursor Protein Shown by Super-Resolution Structured Illumination Imaging

**Figure 7:**
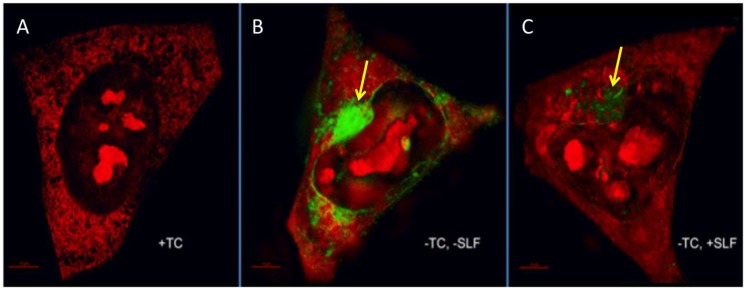
Super-resolution structured illumination (SIM) images of intraneuronal Aβ stained with the amyloid dye FSB (green). In all images, MC65 neuronal cells were stained with propidium iodide (red). No FSB signal is observed for cells grown in the absence of APP induction (+TC, panel **A**). For cells induced for APP expression, a large intracellular Aβ accumulation is apparent (green signal, panel **B**), which leads to a distortion of the nuclear membrane (yellow arrow). When SLF is included in the APP-induced MC65 culture, the Aβ stain signal is greatly reduced (**C**). Scale bar represents 1 μm.

To investigate the specific sub-cellular localization of Aβ deposition and SLF’s role in modulating this process, we cultured MC65 neurons expressing the APP protein without TC for 24 hours in the presence of SLF or vehicle. We assessed Aβ deposits by super-resolution microscopy using the FSB stain, which is a known amyloid dye (Figure 7) [34,35]. Cells grown in the presence of TC served as controls (Figure 7A). In cells overexpressing the APP protein, there is significant FSB staining of accumulated intracellular Aβ (green staining, Figure 7B). The bulk of the deposits surround the nucleus and appear to distort and protrude into the nuclear membrane (Figure 7B). As shown in Figure 7C, there is a greatly reduced FSB signal in cells treated with SLF, which indicates that SLF effectively blocks intracellular Aβ accumulation in cells overexpressing APP.

### 2.5. SLF Decreases the Formation of Intracellular AβO in a Human Neuroblastoma Cell Line Overexpressing the Amyloid Precursor Protein Detected by the Oligomer-Specific Antibody

**Figure 8:**
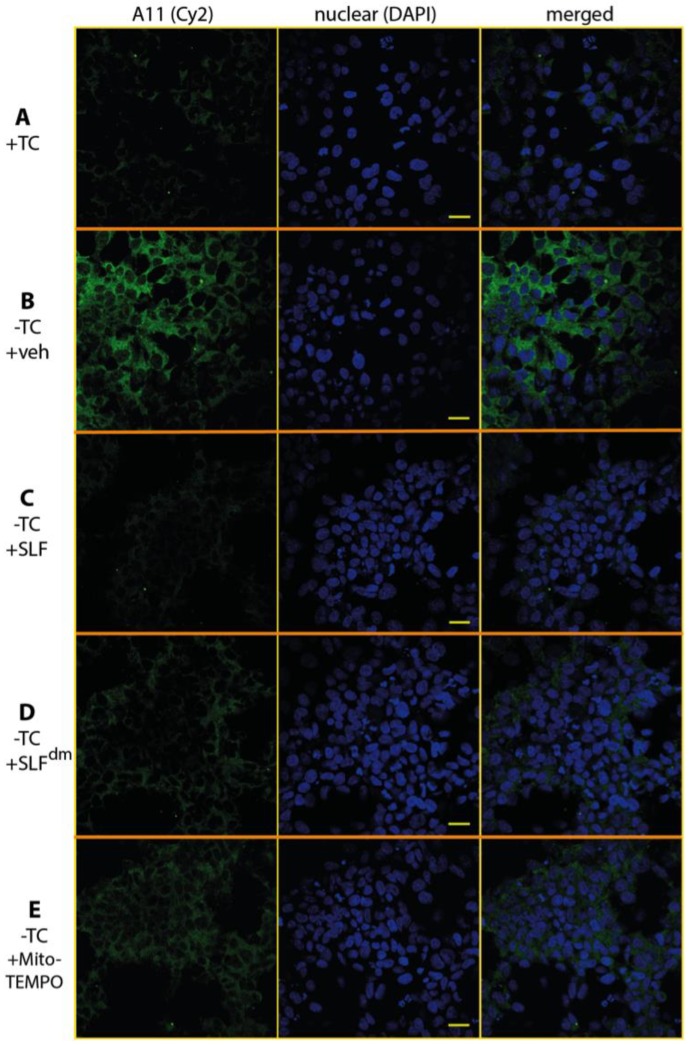
SLF greatly reduces the presence of intraneuronal oligomeric Aβ (AβO) in MC65 cells overexpressing APP. The presence of AβO was detected by the oligomer-specific antibody A11 (indicated by green Cy2 signal) in MC65 cells examined by confocal microscopy. In the absence of APP induction, there is no detection of intraneuronal AβO (**A**). With overexpression of APP, AβO staining is clearly evident (**B**). Addition of SLF to MC65 cells overexpressing APP results in a dramatic decrease in AβO (**C**). Relative to SLF, the ability of diamagnetic SLF to reduce AβO is markedly less (**D**), which suggests that ROS promotes Aβ generation and/or AβO formation. Row (**E**) shows that MitoTEMPO attenuates AβO only partially, which is consistent with the notion of the synergistic action of SLF (conformational effects and targeted antioxidant). In addition to the Cy2 images (left column), images for the DAPI nuclear stain (middle column) and merged DAPI-Cy2 images (right column) are shown. The scale bar represents 20 μm.

**Figure 9:**
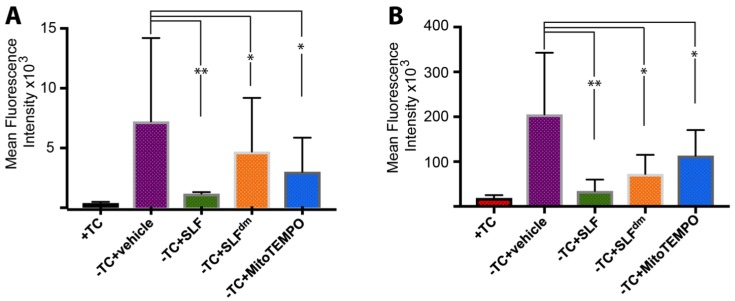
Quantification of the mean fluorescence intensity signal in confocal images of MC65 cells stained with the anti-AβO antibody A11 (panel **A**, see Figure 8) or the generic anti-Aβ antibody 4G8 (for total Aβ, panel **B**, see Figure 10). The effect of SLF, SLF^dm^, and MitoTEMPO co-addition on the intracellular accumulation of Aβ in APP-induced MC65 cells is given by the green, orange, and blue bars, respectively, and is compared to the −TC vehicle control. Statistical analysis of fluorescence intensity by one-way ANOVA gives * *p* < 0.01, ** *p* < 0.001, *n* = 3. Error bars represent the standard error, which is described in the Methods section.

The oligomer-specific antibody A11 provides a powerful tool to detect the presence of Aβ in its toxic, oligomeric state [36,37]. A11 also reacts with oligomeric forms of other amyloidogenic peptides such that it recognizes a conformation specific to pathogenic misfolded proteins. To specifically probe the effect of SLF on oligomeric Aβ (AβO), MC65 neurons overexpressing the APP protein were cultured for 24 hours in the presence of SLF or vehicle, stained with A11 antibody, and examined by confocal microscopy (Figure 8). In the absence of APP induction, no A11 signal is detected (Figure 8A). Upon induction of the Aβ precursor, a clear perinuclear signal for the A11 antibody is evident (green staining, Figure 8B), which shows that much of the accumulated intracellular Aβ is in the oligomeric state. As shown in Figure 8C, SLF treatment of APP-induced MC65 cells effectively removes all A11 staining relative to the non-induced control. SLF lacking its catalytic antioxidant functionality (SLF^dm^) is less efficient in blocking AβO accumulation (Figure 8D), although it performs better than the nitroxide antioxidant alone (Figure 8E). Quantification of the green fluorescent signal of the anti-AβO antibody from confocal images of cultured cells is shown in Figure 9A. SLF shows a large decrease (about seven-fold) in fluorescence intensity from the anti-AβO antibody versus cells treated with vehicle. SLF^dm^ and MitoTEMPO showed approximately a three-fold decrease and about a five-fold decrease in A11 intensity versus treatment with the vehicle alone, respectively (Figure 9A). The intensity of Aβ staining in APP-induced cells without protective treatment was highly variable, which is indicated by the error bars in Figure 9. This can be attributed to uneven localization and aggregation and trafficking patterns of Aβ within the highly stressed cells.

### 2.6. SLF Decreases Accumulation of Total Intracellular Aβ in a Human Neuroblastoma Cell Line Overexpressing the Amyloid Precursor Protein

**Figure 10:**
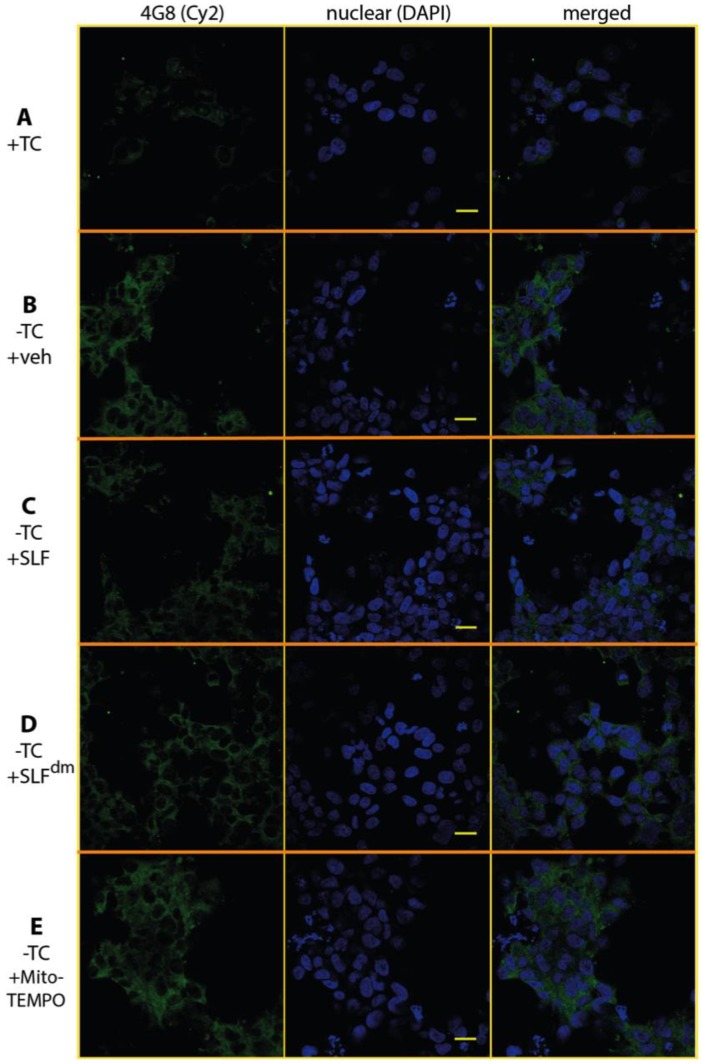
SLF reduces the presence of total intraneuronal Aβ in MC65 cells overexpressing APP. The presence of Aβ was detected by the conformational-independent antibody 4G8, which recognizes all forms of Aβ. Bound 4G8 antibody is indicated by the green Cy2 signal in the MC65 cells examined by confocal microscopy. In the absence of APP induction, there is only slight staining by 4G8 (**A**). With overexpression of APP, Aβ staining is clearly evident (**B**). Addition of SLF to MC65 cells overexpressing APP results in a dramatic decrease in the total Aβ (**C**). Relative to SLF, the ability of diamagnetic SLF to reduce total Aβ is markedly less (**D**), which suggests that ROS promotes Aβ generation and/or inhibits Aβ clearance. Row (**E**) shows that MitoTEMPO attenuates total Aβ slightly better than SLF^dm^, which supports the notion that ROS levels significantly affect Aβ metabolism. The left column is the Cy2 stain, the middle column is the DAPI nuclear stain, and the right column is a merging of the first two columns. Scale bar represents 20 μm.

Intracellular Aβ exists as a dynamic mix of soluble oligomers of various sizes with the small intracellular soluble oligomers considered the most toxic form of Aβ [38]. In order to test whether SLF decreases the total Aβ load within the cells, MC65 neurons expressing APP protein were stained with the 4G8 antibody, which recognizes all conformational states of the peptide. Induced (−TC) cells were treated with vehicle or SLF for 24 hours, stained with the 4G8 antibody for total Aβ levels, and imaged by confocal microscopy (Figure 10). As shown in Figure 10C, SLF substantially lowers total Aβ relative to the vehicle control (Figure 10B). Therefore, the ability of SLF to lower AβO levels (Figure 8) can, in part, be attributed to improved cellular clearance of the peptide generated by APP overexpression. Similarly to their effects on AβO levels, SLF^dm^ and MitoTEMPO treatment also lower total Aβ levels, but to a lesser extent than the bifunctional SLF. Quantification of 4G8 intensity (Figure 8, green signal) is shown in Figure 9B. Regarding their effects on total Aβ, SLF^dm^ outperforms MitoTEMPO, which may indicate that antioxidant functionality is more significant in modulating the formation of AβO while the clearance of total Aβ benefits more from the conformational effects caused by the docking moiety of SLF.

### 2.7. SLF Decreases Uptake of Aβ in Cultured Neurons Treated with Exogenous Aβ

**Figure 11:**
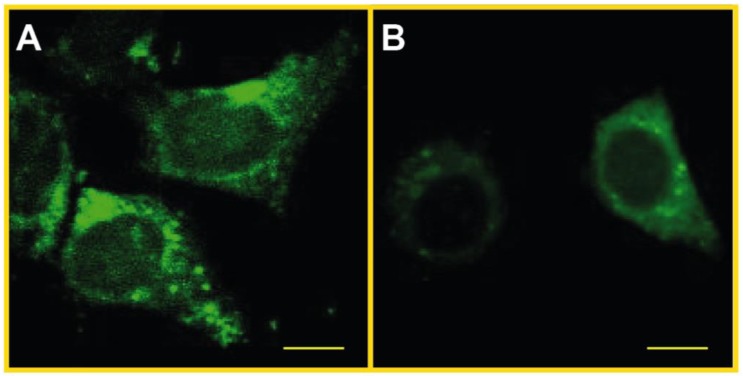
Confocal microscopy of N2a cells stained with the amyloid dye FSB with exogenous Aβ treatment. Panel (**A**) shows cells stained with FSB 24 hours after the addition of 1 μM Aβ. Panel (**B**) shows cells that were treated with 1 μM Aβ + 2 μM SLF and stained with FSB 24 hours later. Bright green dots in (**A**) show a cytoplasmic, perinuclear distribution indicative of intracellular uptake of exogenous Aβ. Scale bars represent 10 μm.

We sought to investigate whether SLF can attenuate intracellular aggregation of exogenous Aβ in N2a neuronal cultures grown in the presence of 1 μM Aβ. We assessed intracellular uptake of exogenous Aβ by measuring the fluorescence intensity of FSB in N2a cells treated with Aβ. As shown in Figure 11, confocal imaging clearly shows a cytoplasmic uptake of exogenous Aβ as evident by the bright green dots around the nucleus and in the cytoplasmic space (Figure 11A). The cytoplasmic distribution of the exogenous Aβ after uptake into the cell matches that of the endogenous Aβ of the MC65 cells post-APP overexpression (green accumulation protruding into the nuclear membrane shown by super-resolution imaging, Figure 7B). The effect of SLF on Aβ uptake was determined by including 2 μM of SLF in the culture medium at the time of the addition of 1 μm Aβ. As shown in Figure 11B, SLF decreases the FSB staining in N2a cells treated with Aβ, which suggests that SLF either inhibits Aβ uptake and/or promotes the peptide’s metabolism within the cell.

### 2.8. SLF Decreases the Presence of AβO in Cultured Neurons Treated with Exogenous Aβ

**Figure 12:**
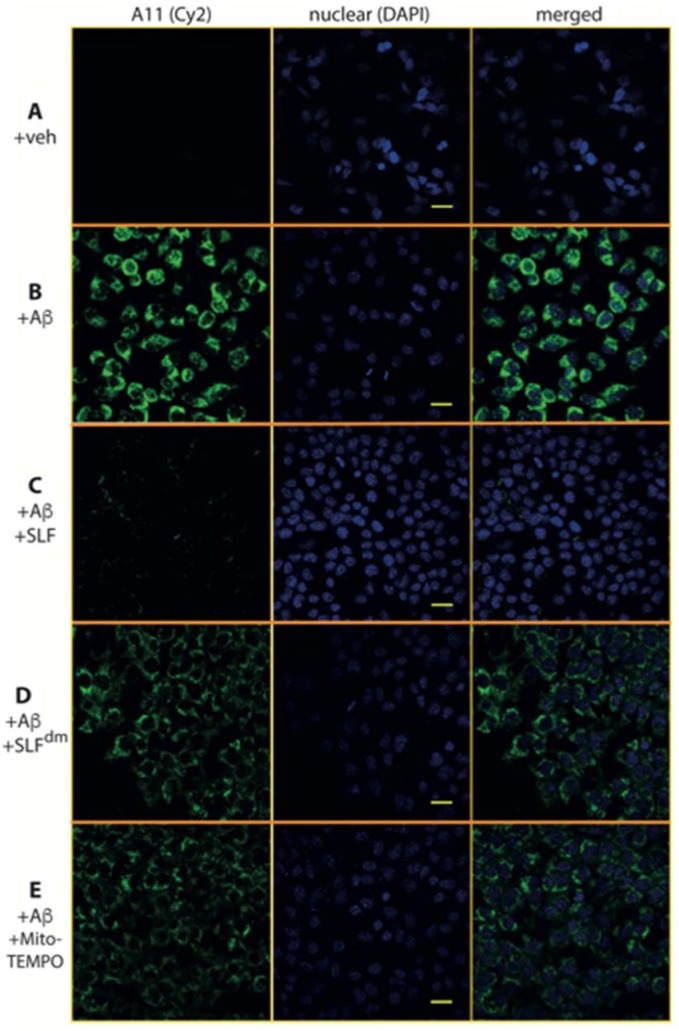
SLF blocks AβO formation of exogenous Aβ added to cultured N2a neurons. Shown are confocal images of N2a cells stained with the oligomer-specific antibody A11 (green signal). A control with only the DMSO vehicle added (no Aβ) is shown in row (**A**). Row (**B**) demonstrates that exogenous Aβ is readily taken up by neurons and adopts the oligomer-specific conformation. Row (**C**) shows the accumulation of imported AβO is blocked by SLF. The significance of the nitroxyl antioxidant functionality in blocking oligomer formation is demonstrated by the diminished ability of SLF^dm^ to inhibit intracellular AβO accumulation (row **D**). Similarly, the antioxidant activity of MitoTEMPO lowers AβO accumulation (row **E**), but not as effectively as the Aβ-targeted SLF compound. The left column is the Cy2 stain, the middle column is the DAPI nuclear stain, and the right column is the merging of the first two columns. Scale bar represents 20 μm.

**Figure 13:**
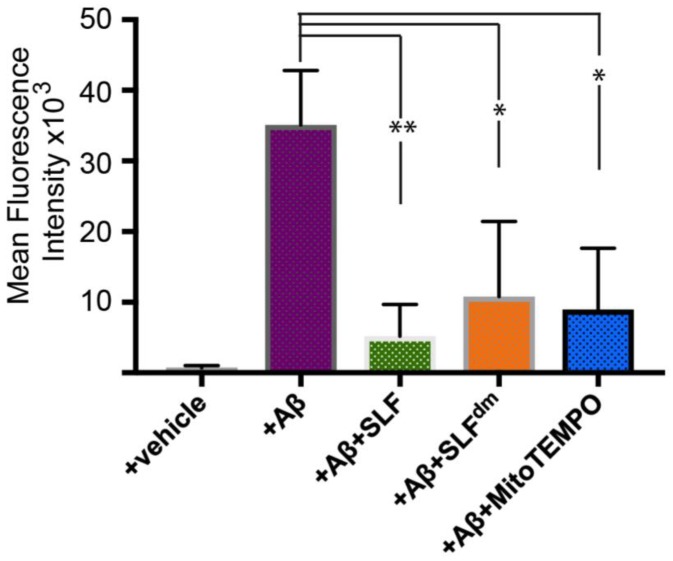
Quantification of mean fluorescence intensity signal in N2a neuronal cells (see Figure 12) treated with exogenous Aβ (1 μM in culture) and stained with anti-AβO antibodies (A11). The effect of SLF, SLF^dm^, and MitoTEMPO co-addition on intracellular accumulation of Aβ is given by the green, orange, and blue bars, respectively, and is compared to the +Aβ group. Statistical analysis of fluorescence intensity by one-way ANOVA gives * *p* < 0.01, ** *p* < 0.001, *n* = 3. Error bars represent the standard error, which is described in the Methods section.

The ability of SLF to specifically reduce oligomeric Aβ in N2a neurons treated with exogenous Aβ was assessed by measuring the fluorescence intensity of the A11 antibody in cells with and without SLF treatment. Significantly elevated fluorescence intensity is indicative of the formation of Aβ oligomers and is noted in the neurons treated with Aβ in the absence of SLF (Figure 12A) while the fluorescence intensity was significantly reduced in neuronal cultures treated with Aβ in the presence of SLF (Figure 12B). To further explore the role of the two moieties of the SLF compound, we compared the isolated effect of the nitroxide component (MitoTEMPO) versus the fluorene component (SLF^dm^) on intracellular AβO levels resulting from exogenous Aβ addition. Although neuronal cells treated with SLF^dm^ (Figure 12D) or MitoTEMPO (Figure 12E) display a reduction in AβO accumulation, the effect is not as pronounced as that of the SLF treatment. Quantification of the fluorescence intensity shows SLF reduces intracellular Aβ aggregation at about seven-fold compared to untreated cells, about two-fold compared to cells treated with MitoTEMPO, and about three-fold compared to cells treated with a diamagnetic version of the fluorene compound (Figure 13).

## 3. Discussion

The amyloid beta (Aβ) peptide is well known for its intrinsically disordered secondary structure and high propensity to form aggregates of various sizes [39,40,41]. While the monomeric form of Aβ is generally considered an antioxidant, metal chelator, and synaptic plasticity enhancer, it is well accepted that the oligomerization of Aβ leads to toxic effects [42,43]. Generated first as a monomer, the oligomerization of Aβ starts intracellularly, which leads to early pathologic oligomeric Aβ species that disrupt neuronal function and lead to decreased cognition and a decline in memory [44]. Disrupting the formation of AβO at the intracellular level is, therefore, crucial for targeting AD pathology during the early stages before permanent damage occurs. 

Oxidative stress remains a common factor connecting several markers for AD pathology both at the onset and during the progression of disease. Therefore, antioxidant activity provides an attractive component for multi-target anti-AD candidates [12]. The role of Aβ in increasing oxidative stress, especially due to its metal binding sites (particularly for Cu^2+^), and its ability to reduce Cu^2+^ and Fe^3+^ to Cu^+^ and Fe^2+^ (generating superoxide anions) have been amply documented using various methods to detect reactive oxidative species [30,31,32]. Therefore, agents capable of localizing anti-ROS activity at Aβ can greatly increase potency. In order to minimize interference with cellular ROS-dependent signaling and endogenous ROS protection pathways, it is becoming increasingly clear that high potency compounds will be especially beneficial to antioxidant approaches [45]. In addition to localizing antioxidant activity at Aβ, the nitroxide is a highly efficient ROS scavenger that can perform in a catalytic fashion [24,25]. 

We have previously reported on the bifunctional ability of SLF to scavenge ROS, decrease Aβ aggregation, and alter the conformation of oligomeric Aβ in vitro [20,21,22,46]. In this study, we investigate how the bifunctionality of SLF can both affect the aggregation dynamics of Aβ and, through the SLF’s nitroxide moiety, maintain ROS homeostasis in cultured neurons. We sought to investigate to what extent the rescued cell survival afforded by SLF is due to its nitroxide antioxidant, fluorene conformational modulator, or the combination of both. The two functional components were evaluated independently by employing a diamagnetic version of SLF (SLF^dm^) that lacks the nitroxyl functional group as well as a potent nitroxyl-based antioxidant (MitoTEMPO) that lacks the ability to engage Aβ and modulate its structure/oligomerization. In neurons challenged by either the overproduction of Aβ (MC65 cells) or by exogenous Aβ addition (N2a cells), SLF provides superior protection compared to SLF^dm^ and MitoTEMPO. With regard to cellular protection, the nitroxide component (represented by the MitoTEMPO treatment) affords greater protection against Aβ addition when compared to SLF^dm^. We propose SLF has the unique capacity to increase neuronal survival in the face of an Aβ challenge by simultaneously decreasing Aβ oligomerization and Aβ-induced oxidative stress. There is ample evidence supporting the presence of a positive feedback cascade between Aβ oligomer formation, ROS generation, and Aβ production with any one of these processes stimulating the rates of the other two, which results in a highly injurious cycle that ultimately leads to neuronal death [16,47,48]. While the two SLF moieties separately act on the specific tasks of oligomerization and ROS scavenging, their results affect each other in the sense that decreased ROS leads to less AβO production and less Aβ results in less oxidative stress.

We then probed the distinct functionalities of SLF by separately investigating its effects on Aβ levels and ROS scavenging. First, we used super resolution microscopy to image the intracellular Aβ load in MC65 cells with and without SLF treatment. The FSB-stained cells show a large accumulation of Aβ. This should be evaluated as total Aβ since FSB stains various forms of amyloid assembly. The high-resolution image reveals a clear perinuclear distribution of Aβ with an apparent distortion of the nuclear membrane. The significance of this distribution in the etiology of AD remains to be investigated. When the APP-induced MC65 cells are treated with SLF, the FSB signal is dramatically reduced. This indicates that SLF not only protects against Aβ toxicity but also affects Aβ metabolism within the cell by decreasing amyloidogenic processing and/or increasing Aβ clearance. Our previous studies [20,21,22] showing that SLF converts AβO into smaller, more stable oligomers may provide a thermodynamic explanation for the enhanced peptide clearance. In this regard, further imaging studies directed towards candidate pathways for SLF-enhanced clearance will be very useful.

To address whether SLF affects the levels of oligomeric Aβ specifically, we carried out confocal analyses of MC65 cells stained with the oligomer-specific antibody A11 as well as the 4G8 antibody, which recognizes all forms of Aβ. Similar to the FSB results described above, untreated MC65 cells induced to overexpress APP reveal a high level of Aβ that appears to outline the nuclear envelope within the cytoplasm. SLF decreases the levels of intracellular AβO as well as total Aβ significantly more than either SLF^dm^ or MitoTEMPO. Similarly, treatment with MitoTEMPO also decreases intracellular AβO content, albeit to a lesser extent. Therefore, while overall protection correlates strongly with the nitroxyl ROS scavenging, the conformational targeting by SLF and SLF^dm^ enhances Aβ clearance over antioxidant activity alone. Nevertheless, the decreased Aβ levels found in MC65 cells with MitoTEMPO treatment align with previous studies showing that oxidative stress promotes intracellular accumulation of Aβ [49,50,51]. A less aggressive amyloid model was also used to evaluate the differential effect of the agents on intracellular AβO accumulation by treating N2a neurons with exogenous Aβ and then staining with the A11 antibody. Confocal images show Aβ is imported into the neurons. However, the amount is substantially less in the presence of SLF. The question of whether SLF is inhibiting Aβ uptake and/or enhancing its clearance remains to be studied.

With respect to the ROS scavenging ability of SLF, our measurements show SLF is a much more efficient scavenger compared to either of its two components taken separately. While SLF containing both the fluorene and nitroxide components significantly decreases oxidative stress, SLF^dm^ has only a mild effect in both the endogenous and exogenous Aβ models. However, the MitoTEMPO treatment shows differing results depending on the situation. Although MitoTEMPO affords less protection than SLF in both cases, it more efficiently protects N2a cells from exogenous Aβ-induced ROS. This might indicate that under a milder Aβ insult, the ROS levels can be strongly attenuated without structural engagement of the Aβ.

These findings support a synergistic effect of the two components of SLF in counteracting both the conformational toxicity of Aβ and its promotion of ROS scavenging, propelling SLF forward as a potential therapeutic candidate for further studies on early progression of AD. Previous findings indicate that the accumulation of Aβ induces oxidative stress and oxidative stress induces the accumulation of Aβ [49]. In light of this link, we postulate the unique bifunctionality of SLF makes it an ideal candidate to pursue as a potentially effective therapy against AD development and progression.

## 4. Materials and Methods

### 4.1. Materials

Spin-labeled fluorene (SLF) and diamagnetic spin-labeled fluorene (SLF^dm^) were synthesized as previously described [52]. Amyloid beta (Aβ) peptide (1–40) was purchased from EZBiolab Inc., Carmel, IN, USA. Hoechst Blue 3342 nuclear stain was purchased from Thermo Fisher, Waltham, MA, USA. CellRox (Deep Red; λ_ex_/λ_em_ 640/665 nm) was purchased from Life Technologies, Waltham, MA, USA. (*E*,*E*)1-Fluoro-2,5-bis-(3-hydroxycarbonyl-4-hydroxy)styrylbenzene (FSB) amyloid stain was purchased from Calbiochem, MilliporeSigma, Burlington, MA, USA. Oligomer A11 polyclonal antibody was purchased from Thermo Fisher, Waltham, MA, USA. The 4G8 anti β-Amyloid 17–24 antibody was purchased from BioLegend, San Diego, CA, USA. The cyanine conjugate (Cy2) donkey anti-goat, secondary antibody was purchased from Jackson Immuno Research, West Grove, PA, USA. The Opti-Minimal Essential Medium (OPTIMEM) was purchased from Invitrogen/Life Technologies. PBS pH 7.4 (-Calcium Chloride, -Magnesium Chloride), Opti-MEM^®^ I Reduced Serum Medium (no phenol red), DMEM (Dulbecco’s modified Eagle’s medium +4.5 g/L Glucose, l-Glutamine, and 110 mg/L Sodium Pyruvate), and Fetal Bovine Serum (FBS) were purchased from Gibco (Carlsbad, CA, USA). 35 mm Glass Bottom Dishes (No. 1.5) were purchased from MatTek, Ashland, MA, USA. Trypan Blue Solution (0.4%) was purchased from Sigma-Aldrich, St. Louis, MO, USA.

### 4.2. Cell Line Model Over-Expressing Intracellular Aβ

We used MC65 cells, a neuronal cell culture line that shows intracellular accumulation of Aβ [29,33,53,54]. The MC65 cells are derived from a human neuroblastoma line with conditional expression of the carboxyl-terminal 99 residues of the amyloid-β precursor protein (APP-C99) under the negative regulation of the suppressor tetracycline (TC) in the culture medium. Proteolysis of APP-C99 by the cellular γ and β secretases generates Aβ. Intracellular Aβ is known to start to accumulate as early as 4 h after TC removal with maximal levels at 24 h. While cell death 3 days after removal of TC was shown to be due to the intracellular accumulation of AβO rather than to the small amounts of secreted Aβ [33], fluorescence microscopy analyses of cells was done at 24 h so that effects of oxidative stress can be acutely attributed to Aβ. However, the MTT assay for cell viability was done at 3 days to allow for the observation of changes in cell morphology. The MC65 cells were cultured in a 75 mL flask in a culture medium composed of a mixture of 50% Dulbecco’s Modified Eagle Medium supplemented with 4.5 mg/mL d-glucose, non-essential amino acids, 1 mM sodium pyruvate, and 10% (*v*/*v*) heat-inactivated fetal bovine serum, supplemented with 0.1 mg/mL tetracycline, 50 IU/mL penicillin, and 50 μg/mL streptomycin. The cultures reached 80% confluence after 24 h. At this point, the cells were harvested and the trypan blue exclusion test was used to determine the count of viable cells for calculations of cell density. Cells were then seeded at a density of 2.5 × 10^5^ cells/well in 96-well plates for the MTT viability assay and in glass bottom culture dishes for the other experiments. Expression of APPC99 in MC65 cells was induced by removing TC from cell culture medium and cells were treated with various treatments described for each experiment. To examine the effects of SLF on exogenous Aβ, we used the Neuro-2a mouse neuroblastoma cell line (N2a). The N2a cells were cultured in the same culture media as the MC65 cells (only without TC in the culture media) in a 75 mL culture flask and reached 80% confluence after 12 h. They were then harvested and processed similarly to MC65 cells. Exogenous Aβ was prepared as described in Section 4.3 and was added to a final concentration of 1 μM in +/− for the same treatments as those of the MC65 cells. For both cell lines, we cultured cells from three different passages on different days. For the fluorescence intensity quantification, we selected three clusters of 30 cells from each image to normalize for the cell number and background intensity. We then used Image J to quantify fluorescence intensity for the CellRox and Cy2 channels.

### 4.3. Preparation of Amyloid-Beta Peptide Solution

Aβ solution was prepared as previously described [20,21,23]. The Aβ peptide was reconstituted in hexa-fluoro-isopropanol (HFIP) and incubated at room temperature on a shaker for 48–72 h until it became clear. Aliquots of 0.1 mg Aβ were placed in a SpeedVac to remove the HFIP and the resulting monomeric peptide film was stored at −80 °C. Immediately before use, the Aβ peptide film was reconstituted in 10 μL fresh DMSO for a stock solution of 1 mM Aβ. The Aβ solution diluted to a final concentration of 1 μM was co-added to the N2a cultured cells treated with either vehicle or 2 μM final concentration of SLF, mitoTEMPO, or diamagnetic fluorene (SLF^dm^). Since Aβ starts to aggregate almost immediately after the addition to the culture media, we introduced the Aβ solution to the N2a cell culture simultaneously with the compounds and incubated at 37 °C for 24 h to let Aβ oligomerize in situ. Although the concentration of the intracellular Aβ is not known, it is likely higher than that of the 1 μM Aβ added to the culture media. We choose the 1:2 Aβ:SLF molar ratio since, at these concentrations, Aβ is expected to be fully saturated with SLF [21,22].

### 4.4. Cell Viability Assay

Cytotoxicity in N2a and MC65 cells cultured and treated as described in Section 4.2 was determined using counts of viable cells 72 h after treatment based on the MTT assay (tetrazolium dye MTT 3-(4,5-dimethylthiazol-2-yl)-2,5-diphenyltetrazolium bromide), which was described previously by Maezawa et al., 2007. Both N2a and MC65 cells were plated at a density of 2.5 × 10^5^ cells/well in a 96-well plate in 200 μL in the same medium described in Section 4.2. The Aβ solution was prepared in phenol red-free (OPTIMEM) and fetal bovine serum-free media as described in Section 4.3, then added to the N2a cell cultures at final concentrations of 1 μM for Aβ and 2 μM for SLF, SLF^dm^, and MitoTEMPO. The MC65 cells were cultured in the same conditions in the presence and absence of TC at 1 μg/mL. The compounds were added to the cultures at 2 μM for SLF, SLF^dm^, and MitoTEMPO for 9 wells and 3 rows. After 24 h of incubation, 150 μL media was removed and 5.5 μL of 2.5 mg/mL MTT (thyazolyl blue compound) was added to each well to a final concentration of 0.25 mg/mL. After 2 h incubation at 37 °C, 100 μL of solubilization buffer (0.1 N HCL in isopropanol) was added to each well and the plates were placed on a shaker at room temperature until blue crystals are dissolved. The plate was read on the plate reader at 560 nm for MTT and 630 nm for baseline subtraction. Cell viability was expressed as the percentage of viable cells.

### 4.5. Cell Culture for the Detection of Intracellular Aβ by Super-Resolution Structured Illumination Imaging

The MC65 neuronal cells were washed extensively and plated at a density of 3.5 × 10^5^ cells/well over 18/18 mm #1.5 Fisherbrand Microscope Cover Glasses in OPTIMEM containing 50 IU/mL penicillin and 50 μg/mL streptomycin without serum and without TC. The neuronal cells were treated with 10 μM SLF or with the vehicle. The control cells were plated in OPTIMEM with TC. After 24 h of incubation at 37 °C, the cultures were washed in PBS pH 7.4 for 15 min × 3 to discard any extracellular Aβ, fixed in 4% paraformaldehyde, and stained for 10 min in 5 μM (*E*,*E*)1-fluoro-2,5-bis-(3-hydroxycarbonyl-4-hydroxy)styrylbenzene (FSB) amyloid stain. After washing in PBS pH 7.4 for 1 h × 3, the cover glasses were mounted with Vectashield Mounting Medium with Propidium Iodide (PI) over microscope slides [34]. 

### 4.6. Super-Resolution Structured Illumination Imaging of FSB-Stained Intracellular Aβ

Super-resolution structured illumination (SIM) imaging was used to obtain images of FSB-stained Aβ inclusions (green) in MC65 cells overexpressing APP protein, which were grown in the presence (+TC) and absence (−TC) of an inducible repressor. Images were taken using a Deltavision OMX v2.0, (Applied Precision, LLC, Issaquah, WA, USA) super-resolution, wide-field, Structured-Illumination fluorescence Microscope (SIM) that uses a 60× magnification and 1.40 NA objective lens immersed in 1.514 refractive index oil. The FSB/PI staining was viewed using excitation lasers at λ_ex_ = 405 nm and 488 nm, respectively. The *Z*-stacked images were reconstructed using the deconvolution software SoftWoRx version 4.1 (GE Healthcare, Marlborough, MA, USA). Reconstructed images were processed using Volocity 5.5.1 (Perkin Elmer Inc., Waltham, MA, USA) to generate 3-D images.

### 4.7. Immunofluorescence Staining for the Accumulation of Intracellular Aβ

MC65 and N2a neurons were cultured on cover slips, treated as described in Section 4.3, incubated at 37 °C for 24 h, washed in untreated culture media for 15 min × 3 to discard any extracellular Aβ, and fixed in 4% paraformaldehyde. To detect the accumulation of intracellular Aβ oligomers, the MC65 and N2a cells were then stained with anti-Aβ oligomer A11 antibodies (1:2000) overnight at 4 °C, and then with Cy2 fluorescent secondary antibodies for 3 h at room temperature. To detect the accumulation of total intracellular Aβ, the MC65 cells were stained with anti-Aβ antibodies 4G8 (1:1000) overnight at 4 °C followed by Cy2 fluorescent secondary antibodies for 3 h at room temperature. Additionally, to detect intracellular uptake of Aβ, the N2a cells treated with exogenous Aβ (1 μM) in the presence and/or absence of SLF (2 μM) for 24 h were stained for 10 min in 5 μM amyloid dye FSB ((*E*,*E*)1-fluoro-2,5-bis-(3-hydroxycarbonyl-4-hydroxy)styrylbenzene). After staining, cells were washed extensively in culture media ×3, fixed in 4% paraformaldehyde for 10 min and imaged [34,35,54].

### 4.8. Detection of Intracellular Aβ Oligomers, Total Aβ, and Intracellular Uptake of Aβ by Confocal Microscopy

Fixed cells were imaged with an Olympus Fluoview FV1000 spectrum confocal microscope (Life Science Solutions, Center Valley, PA, USA). Each individual field was imaged using an ×40 objective. Single plane confocal scans of the cultured MC65 neuronal cells and N2a cells areas were taken via sequential scanning mode. Fluorescence intensity comparison was then used to determine differences between the accumulations of intracellular Aβ under various treatments. 

### 4.9. Detection of the Intracellular Oxidative Stress Signal by Confocal Microscopy

Treated and control MC65 and N2a cells were gently washed with untreated culture medium and incubated for 30 min with the ROS detection reagent CellROX, which is a fluorogenic probe that when oxidized develops a red fluorescent signal seen around the nuclei of unprotected cells. At 20 min, the cells were treated for the remaining 10 min with a Hoechst Blue 3342 nuclear stain, gently washed for 15 min × 3 with untreated culture medium, and imaged immediately. The images of CellROX staining were collected on an Olympus FV1000 spectrum confocal microscope. Each individual field was imaged using an ×40 objective. Single plane confocal scans of the cultured neuronal cell areas were taken through the sequential scanning mode using diode excitation lasers of 653 nm for CellROX Deep Red (λ_exe_/λ_em_ = 640/665 nm) and 559 nm excitation laser for Cy2 (λ_exe_/λ_em_ = 492/510 nm). Intensity comparison of the ROS signal was then performed to compare Aβ-induced oxidative stress. 

### 4.10. Statistical Analysis and Quantification of Immunohistochemical Staining

Statistical significance between groups of cells under different treatments was determined by an ordinary one-way ANOVA test using GraphPad Prism version 7.0c for MAC OS X, GraphPad Software, La Jolla, CA, USA, where *p* value from the ANOVA is reported as a result of the Brown-Forsythe test and is considered significant if <0.05. All data were expressed as the mean ± SEM from multiple measurements. For each measured condition in MTT assays, *n* = 12. For fluorescence intensity measurements, *n* = 3, and multiple ROIs were measured from groups of 30 cells with 3 areas of each image obtained from 3 different dishes of cells for each group. Each dish was cultured from a different stock of a different passage (12–14).

The images of the CellRox and Aβ-stained cells were transformed to 8-bit gray scale and fluorescence intensity was analyzed with Image J, FIJI (version 2.0.0-rc-68/1.52e, open-source platform for biological image analysis) for MAC OS X, using the particle analysis function [55]. One-way ANOVA followed by a Tukey multiple comparisons test was performed to compare mean fluorescence intensity between the treatment groups using the GraphPad Prism version 7.0c for MAC OS X, GraphPad Software, La Jolla, CA, USA. Triplicate measurements were done in 3 randomly selected areas of each of the cell culture fields with a background correction. All data were expressed as the mean ± SEM.
